# Modelling of Infectious Diseases for Providing Signal of Epidemics: A Measles Case Study in Bangladesh

**DOI:** 10.3329/jhpn.v29i6.9893

**Published:** 2011-12

**Authors:** Sifat Sharmin, Israt Rayhan

**Affiliations:** Institute of Statistical Research and Training, University of Dhaka, Ramna, Dhaka 1000, Bangladesh

**Keywords:** Communicable diseases, Disease models, Disease outbreaks, Seasonal autoregressive integrated moving average model, Statistical process-control charts, Bangladesh

## Abstract

The detection of unusual patterns in the occurrence of diseases is an important challenge to health workers interested in early identification of epidemics. The objective of this study was to provide an early signal of infectious disease epidemics by analyzing the disease dynamics. A two-stage monitoring system was applied, which consists of univariate Box-Jenkins model or autoregressive integrated moving average model and subsequent tracking signals from several statistical process-control charts. The analyses were illustrated on January 2000–August 2009 national measles data reported monthly to the Expanded Programme on Immunization (EPI) in Bangladesh. The results of this empirical study revealed that the most adequate model for the occurrences of measles in Bangladesh was the seasonal autoregressive integrated moving average (3, 1, 0) (0, 1, 1)_12_ model, and the statistical process-control charts detected no measles epidemics during September 2007–August 2009. The two-stage monitoring system performed well to capture the measles dynamics in Bangladesh without detection of an epidemic because of high measles-vaccination coverage.

## INTRODUCTION

Infectious diseases are a major cause of misery, sickness, and death in humans and animals worldwide. Control and prevention are, therefore, an important task from both a humane and an economic point of view. Aberrations in usual distribution of an incidence of disease may provide an early signal of an epidemic of a disease in time or space. An early identification of epidemics of infectious diseases is an important first step towards implementing effective interventions and reducing the resulting mortality and morbidity in human populations. Thus, the detection of unusual patterns in the occurrence of diseases is an important challenge to public-health surveillance. Dynamic modelling of infectious diseases has contributed greatly to this end (1). Surveillance data are usually used for such a modelling purpose, although surveillance by notification has a problem to underestimate true case numbers. Under-notification leads to an underestimation of the burden of disease and hinders the implementation of appropriate prevention and control strategies.

Box-Jenkins or autoregressive integrated moving average (ARIMA) modelling is such a technique which enables the dependency structure embedded in infectious disease time-series data to be modelled and has potential research applications in studies of the disease dynamics (2). Statistical process control (SPC) is another branch of statistics that combines rigorous time series analysis methods with graphical presentation of data, often yielding insights into the data more quickly and in a way more understandable to lay decision-makers. Understanding the characteristics of the control charts can lead to timely detection of changes in the levels of disease series and subsequent timely public-health actions to decrease unnecessary morbidity and mortality (3,4).

Trottier *et al*. tried to characterize the basic statistical dynamics of childhood infectious diseases to obtain a better understanding of different disease patterns and explored the possibility of classifying the infections by their corresponding time series structure using the ARIMA modelling approach (5). Williamson and Hudson successfully identified and fitted the seasonal autoregressive integrated moving average (SARIMA) model for hepatitis A, hepatitis B, hepatitis non-A-non-B, legionellosis, malaria, meningococcal infections, and tuberculosis (6). Helfenstein was the first to showthat the ARIMA models can be used for forecasting and detecting the relationships between different disease series (7). A few authors have, however, used Box-Jenkins modelling to forecast the mortality incidence of pneumonia and influenza in the United States (8,9) or infectious diseases in Canada (10). VanBrackle and Williamson demonstrated how the monitoring system with several control charts detects spike, step, and trend changes of varying sizes in the disease series (11).

This study employed a two-stage monitoring system based on the successful integration of (a) detection of aberrations in the occurrence of diseases from the historical pattern and (b) providing signal of the epidemics using the ARIMA model and SPC charts. To show the applicability and effectiveness of this monitoring system in practical application, we conducted an illustration for detecting the epidemic situations of measles in Bangladesh.

Despite the development of an effective vaccine in the 1960s, measles is a leading vaccine-preventable killer of children in the developing world. Currently, the burden of disease in developing countries of Asia results principally from communicable diseases, such as measles, malaria, pneumonia, diarrhoea, and nutritional disorders. In Bangladesh, the disease still remains the fifth leading cause of death among children aged less than five years. According to the World Health Organization, in Bangladesh alone, 20,000 children die every year due to measles, i.e. 54 children die every day (12). In 2002, measles accounted for 3% of the disability-adjusted life-years lost among the top 10 causes of death in Bangladesh (13). The Government of Bangladesh has prioritized universal immunization of children against this vaccine-preventable disease. According to the Bangladesh Demographic and Health Survey (BDHS) 2007, about 83% of children have received measles vaccine (14). This is an increase from 76% in the BDHS 2004 (15) and 71% in the BDHS 2000 (16). Despite the high vaccination coverage, global immunization experts warn of resurgence in measles-associated deaths if vaccination efforts are not sustained.

## MATERIALS AND METHODS

Analyses were performed on a univariate time-series of monthly national measles cases during January 2000–August 2009 in Bangladesh; the data were obtained from the EPI.

The Box–Jenkins ARIMA modelling strategy was employed in the first stage of this study to find the best fit of measles time-series data because this technique is designed to properly handle the autocorrelation issues which arise because of the trend, seasonal and cyclical components present in infectious disease data (2). SARIMA is an extension of ARIMA where seasonality is accommodated with seasonal differencing.

A time series {x_t_} is a SARIMA (*p*, *d*, *q*) *(P,D,Q)_S_* process with period *S* if *d* and *D* are non-negative integers and if the differenced series *Y_t_* = (1-B) (1-B^s^)^D^ x_t_ is a stationary ARMA process defined by the expression:

Ф(B)⊖(B^s^)Y_t_ = ⊖(B)⊖(B^s^)e_t_

where B is the backshift operator, and e_t_ is a sequence of normally and identically distributed random shocks with mean zero and constant variance.

The parameter *p* and *P* represent the non-seasonal and seasonal autoregressive polynomial order respectively, and the parameter *q* and *Q* represent the non-seasonal and seasonal moving average polynomial order respectively. The parameter *d* represents the order of normal differencing, and the parameter *D* represents the order of seasonal differencing.

The modelling procedure begins by choosing an appropriate ARIMA model through the extensive model identification and selection steps, which is done with the help of the autocorrelation and partial autocorrelation functions. Parameters of the selected model are then estimated using maximum likelihood estimation. Finally, residuals of the estimated model are examined in the diagnostic checking stage by performing a Ljung-Box test or plotting autocorrelation and partial autocorrelation of the residuals. While this model has the advantage of accurate forecasting over short periods, it also has the limitation that at least 50, preferably 100, observations or more should be used (17).

In the second stage of this study, the following three types of SPC charts were used for tracking the signal of epidemics: (a) Shewhart, (b) moving average, and (c) exponentially-weighted moving average (EWMA).

The Shewhart control chart is a graphical and analytical tool for deciding whether a process is in a state of statistical control. It detects large deviations (1.5 to 2.0 standard deviations or greater) from the previous stable pattern very quickly but is not effective in detecting smaller shifts. This chart considers only the last-plotted individual point, thus ignoring information about the process in previous observations.

The moving average (MA) control chart uses the MA of observations of the process as the control statistic and is more effective than the Shewhart chart in detecting small process shifts in the level of the process. For the MA control chart, the MA of span *m*, the average of the last *m* points, is plotted rather than the individual point.

The EWMA is a weighted average of observations; it is less sensitive to the assumption of normality and, therefore, provides more flexibility in its application to monitoring problems. It combines historical data to give less weight to data as they get older and is more sensitive in detecting small shifts in a process since they use information from a long sequence of samples. The upper and lower control limit (UCL and LCL) lines of the three charts display the range of expected variations in their summary statistics. Points above the UCL or below the LCL lines are out-of-control points while points inside the UCL and LCL are in-control points (18).

All analyses were performed using the EViews (version 5), SAS/QC^R^ (version 9.0) and MATLAB (version 7.0.0.19920(R14)) software packages.

## RESULTS

The analyses started by applying the Box-Jenkins modelling strategy for the identification, estimation, diagnostic checking, and forecasting of the univariate time-series data on monthly measles cases in Bangladesh.

The historical data were divided into two periods: one for model estimation and one for forecasting, model evaluation, and testing. The data of the January 2000–August 2007 period were used for constructing a SARIMA model and those of the September 2007–August 2009 period were then used for validating the model.

**Fig. 1.**
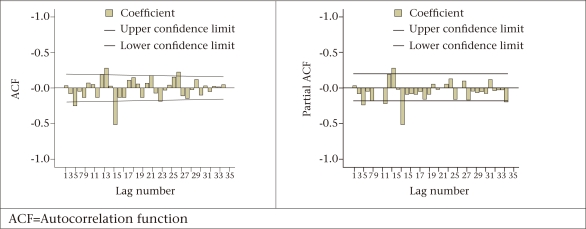
Correlogram and partial correlogram, first differences of measles of order 1 and 12, Bangladesh, 2000 to 2007

The measles cases exhibited periodicity of length 12. That is why, the first differencing of order 1 and 12 was performed to achieve stationarity (Fig. 1 depicts the first differences of order 1 and 12). The sample autocorrelation and partial autocorrelation functions (ACF and PACF) in Figure 1 allowed one to identify an appropriate SARMA form to model the stationary series. Indeed, the decaying PACF after lag 3 suggested an autoregressive (AR) component of order 3. Also, the ACF indicated that a seasonal MA component of order 1 would be appropriate since a sole significant spike at lag 12 was detected. Thus, a SARIMA (3, 1, 0) (0, 1, 1)_12_ model was suggested as appropriate for the univariate measles time-series data in Bangladesh.

**Fig. 2.**
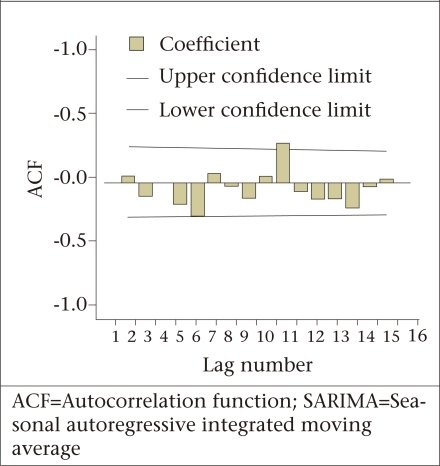
Correlogram of residuals obtained from the SARIMA (3, 1, 0) (0, 1, 1)_12_ model

The residual ACF (Fig. 2) of the estimated SARIMA (3, 1, 0) (0, 1, 1)_12_ model exhibited no specific pattern and only one significant spike. It was, therefore, concluded that the series of the residuals behaves like white noise and that the model fitted best to the data.

**Fig. 3.**
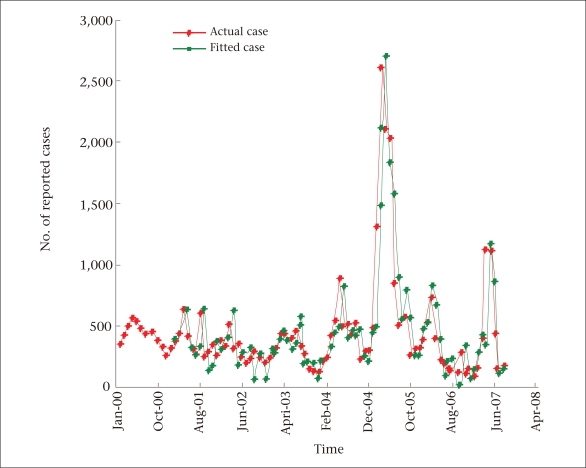
Observed vs fitted values of measles

The plot of the observed versus fitted values (Fig. 3) indicates that the model provided an excellent fit to the data.

**Fig. 4.**
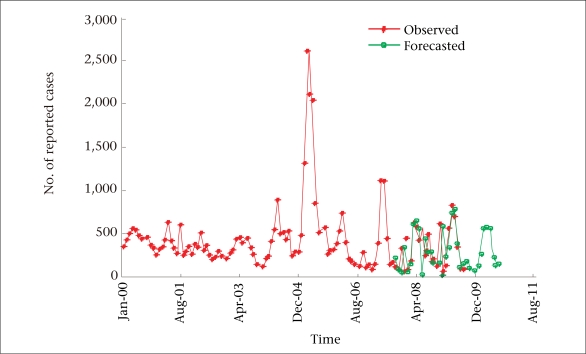
Observed vs forecasted values of measles

The SARIMA (3, 1, 0) (0, 1, 1)_12_ model was then used for forecasting monthly measles cases for the next 36 months: September 2007–August  2010. The one-month-ahead, rolling forecasts for each of the 36 months were calculated, which closely mirrors the observed values available for the first 24 months: September 2007–August 2009 (Fig. 4).

Thus, on the basis of in-sample and out-of-sample forecasts, we concluded that the model had sufficient predictive power, and the findings are well in line with those of other studies (6-10).

**Fig. 5.**
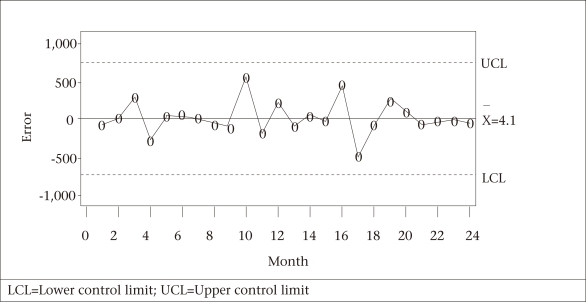
Shewhart control chart for the number of reported cases of measles in Bangladesh by month

**Fig. 6.**
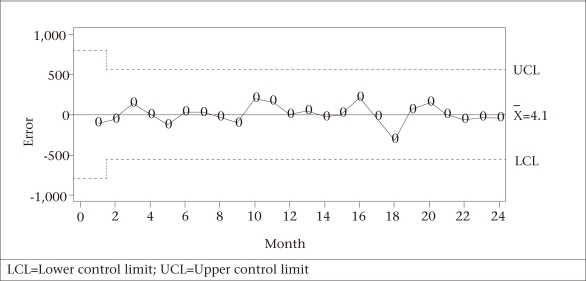
Moving average control chart for the number of reported cases of measles in Bangladesh by month

**Fig. 7.**
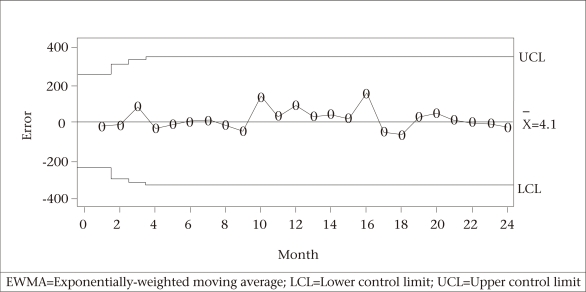
EWMA control chart for the number of reported cases of measles in Bangladesh by month

The three SPC charts—Shewhart and MA (with span of size 2) and EWMA— executed to monitor and identify aberrations in the forecast errors for the last 24 months (September 2007–August 2009) are shown in Figure 5-7. None of the three charts detected any significantly high values for the 24-month measles forecasted data, implying that there was no significant deviation from the forecasted or expected values of measles counts for those 24 months.

## DISCUSSION

This study was an attempt to detect aberrations in the occurrence of infectious diseases from the historical pattern using the Box-Jenkins modelling approach and then using the SPC charts as an aid to the detection of epidemics so that efficient interventions can be taken to prevent further cases and to get the disease under control.

Box-Jenkins modelling, also called the autoregressive integrated moving average model, can be used for making decision in disease surveillance, prediction, and risk management as suggested by previous studies. In the first part of this study, the Box-Jenkins modelling approach was used. The empirical results suggest the SARIMA (3, 1, 0) (0, 1, 1)_12_ model as the best and accurate one, which successfully identified the pattern of the measles dynamics in Bangladesh. Our findings are well in line with those of other studies (6-10). The first suggestion to apply the Box-Jenkins method to incidence data for infectious diseases was given by Armitage (19). Choi and Thacker also declared that the Box-Jenkins method provides a better forecast than the traditional method for case notifications of infectious diseases (8,9).

One of the strengths of our study was to employ several SPC charts to detect aberrations in the occurrence of disease from the historical pattern and, thus, to provide an early signal of epidemics. The control charts applied to the residuals of one-step-ahead forecasts based on the Box-Jenkins models of reported cases of measles exhibited no irregular behaviour. This finding pointed out the fact that no significant change in the trend of the occurrence of measles in Bangladesh had taken place during the prediction period (September 2007–August 2009). Benneyan *et al.* illustrated how the control charts might be able to detect statistically significant signals from the patterns in data more quickly than with traditional statistical methods (20).

To help manage a disease, it is important to discover the likely outcome of an epidemic, and this modelling approach can provide timely signalling of the onset of measles epidemics in Bangladesh by notifying statistically significant departures in the occurrence of measles from what is expected based on its historical incidence. Thus, the monitoring system, which was a combination or blend of forecasting strategies with specific control charts, can be used for understanding the behaviour of diseases over time and can help health programme planners to implement effective interventions to reduce the occurrences of disease and to cut down the resulting mortality and morbidity among the people.
